# Vice and Impoverishment: Two Perfectionist Bads

**DOI:** 10.1007/s10790-022-09906-5

**Published:** 2022-09-21

**Authors:** David Machek

**Affiliations:** Institut für Philosophie, Universität Bern, Länggassstrasse 49a, 3012 Bern, Switzerland

## Introduction

Recent years have seen an impressive development of philosophical theories of human well- being or welfare. A philosophical theory of well-being aims at an account of what makes my life fare well, or go well *for* me. According to a standard classification, there are three major approaches to well-being.^1^ Crudely put, we can say that on the hedonist theory, well-being is determined by presence of pleasure and its preponderance over pain; according to the desire-fulfilment view, well-being depends on having one’s desires satisfied; and according to the objective-list theory, one fares well if one’s life is rich in certain objective goods, such as knowledge, friendship or achievement. There is also a theory known as perfectionism, which is sometimes understood as a version of the objective-list theory and sometimes rather as a *sui generis* approach to well-being.^2^

What perfectionism has in common with the objective-list theory, in contrast to the hedonist and desire-fulfilment theories, is that well-being derives from objective values or achievements rather than from subjective feelings or attitudes. The distinctive claim of perfectionism, which constitutes its advantage but also its potential weakness, is that it provides a principled criterion for the specific goods that should be included on the list and a justification for why this is the case. What is good for us must be determined by who we are; insofar as we are human beings, and humans are defined by certain capacities that are essential, necessary or characteristic for them, things that are good for us as individuals amount to, or derive from, the goodness of these capacities.^3^ So, perfectionism defines well-being in terms perfection or development of these capacities and their exercise. Typically, these capacities include, first and foremost, practical and theoretical rationality, but may also include a diverse range of emotional and bodily capacities.

Perfectionism has been criticised on various grounds. Besides misgivings about perfectionist elitism or justification for, and explanatory power of, the underlying account of human nature, perhaps most serious and most often raised objection is that perfectionism leads to a dubious “gap between well-being and subjective experience.” 4 One consequence of this gap is that perfectionism has difficulties to account for obvious badness of certain kinds of subjective experiences, such as pain; the converse aspect is that it struggles to explain why some elements of objective perfection should be good for me, or why elements of objective anti-perfection should be bad for me.

This article turns to yet another alleged weak point of perfectionism, namely that it lacks an adequate account of prudential disvalue, or what is bad for us, in and of itself. Given that perfectionism defines prudential goodness as a development or perfection of capacities, it seems that what is bad can only be defined as a lack of development, or, at best, as a diminishment or loss of capacities, or inhibition of their exercise.^5^ But these shortcomings are merely absences of what is good, rather than robust, genuine bads. Richard Kraut talks about “unflourishing” caused by uncomfortable sensations such as hunger but it is not clear, precisely, why these experiences should be bad on perfectionist grounds.^6^ Insofar as we think that “any reasonable theory of well-being will include not only intrinsically positive elements but also intrinsically negative ones as well,” this alleged lack of robust disvalue would count against perfectionism as a compelling theory of well-being.^7^ (Whether each and every plausible theory of well-being must have an account of robust badness is potentially controversial. This article does not aspire to settle this question but argues that perfectionism can be vindicated *even if* this requirement is in place.)

Recently, Gwen Bradford has undertaken a sophisticated attempt to vindicate perfectionism on this front by offering an account of perfectionist bad as a “malfillment” of capacities.^8^ The objective of this article is to engage with this proposal and on that basis offer an alternative account of perfectionist badness. I argue, firstly, that Bradford’s proposal as it stands invites several objections that can, to some extent at least, be remedied by appending qualifications; secondly, that these qualifications pave the way for acknowledging the existence of other more fundamental perfectionist bads, one that Bradford all-too-quickly dismisses, and another one she does not consider at all. This account of perfectionist bads will be closely inspired by Aristotle, the alleged “grandfather” of perfectionism.^9^ Insofar as several contemporary perfectionist draw on Aristotle, in one way or another, it stands to reason to mine Aristotle more thoroughly than has so far been the case for a possible account of robust perfectionist bads. The historical links between contemporary perfectionism and Aristotle’s eudaimonism raise difficult questions about whether Aristotle’s ethical theory can be regarded as proto- perfectionist, as well as about overlaps and differences between perfectionism and eudaimonism as theories of well-being. So, for instance, Dan Russell construes his eudaimonistic theory as Aristotelian, but wants to distinguish it sharply from perfectionism, which he regards as a “non-starter” (Russell 2012: 45-53).^10^ In drawing on some elements of Aristotle’s theory to vindicate perfectionism, I remain non-committal about the precise nature between (Aristotelian) eudaimonism and perfectionism. What matters here is merely that some crucial elements of Aristotle’s eudaimonism, such as notions of virtue and vice, function without difficulties in the perfectionist framework: if virtues are forms of perfection of the human nature, then clearly they are conducive to well-being in the perfectionist sense.^11^

I shall argue that whereas “malfillment” does fit the bill for what perfectionist bad should look like, there are two other, and more fundamental, perfectionist bads: moral and epistemic *vice*; and *impoverishment*, which includes certain forms of deprivation, namely loss-related and waste-related deprivations of perfectionist good that constitute a net negative value. Vice is bad because certain potentially worthwhile capacities and activities have acquired a bad or perverted quality; impoverishments are bad because some worthwhile activities and capacities are, in peculiarly qualified ways, absent from one’s life. Vice is not a mere privation, insofar as it presupposes a developed, but perverted, form of rationality. As such, it needs to be distinguished from a range of privative conditions in which rational capacities are non-developed, diminished or lost, as well as from conditions where these capacities are intact but their exercise is impeded or inhibited. The intrinsic badness of some forms of impoverishments derives from the fact that the privation they entail is indexed, or relative to, to another, richer state. The robust badness of these special kinds of impoverishment is attested by the intuitive view that some forms of extremely impoverished lives are not worth living, all things considered, and that death may in fact be preferable to these kinds of lives. Since death is an absolute privation, and some impoverished lives are worse than death, whatever makes these lives worse than death must be worse than privation.

I start with a discussion of Bradford’s proposal (section 2), then turn briefly to Aristotle’s account of lives not worth living (section 3) and spell out my account of vice (section 4) and impoverishment (section 5) as robust perfectionist bads. In the concluding part (section 6), I propose what vice and impoverishment as two major perfectionist bads have in common.

## The Malfillment-Account

Bradford begins her exposition of the malfillment view by rejecting what she calls the “inhibition” approach to perfectionist bads. On the inhibition view, the bad amounts to a condition in which exercise of our capacities is “inhibited, thwarted or diminished.”^12^

It is not that we are simply not exercising our capacities, but that we are prevented from doing so. Bradford notes correctly that this view presupposes that the inhibition must be relative to a state in which capacities can be exercised more freely but suggests that it is this indexicality that ultimately makes inhibition an implausible candidate for a perfectionist bad.

She first considers the case of Einstein whose rational capacities are twice as good as those of an average person at their best, but are inhibited so that they can only be exercised to a half of what they would be otherwise. If inhibition were a genuine bad, then the inhibited Einstein would have to fare worse than an uninhibited average person, even though their actual performance would actually be at the same level of perfection. But then it is counter-intuitive to say that Einstein is faring badly, especially when we grant that the average person is faring quite fine. As an alternative, Bradford considers the possibility that the inhibited state could be indexed to what she calls a baseline standard, e.g. an IQ-level below which one would be in a mentally deranged condition, given the intellectual capacities characteristic for humans. Eventually, she dismisses the inhibition approach on the grounds that inhibition cannot be robustly bad because in some situations it is instrumentally good, such as when it promotes the development of our capacities by confronting them with a healthy challenge.

Perfectionist theories typically commit to what Bradford calls the “twofold scheme,” or two axiological levels: capacity, e.g. capacity for having beliefs, and activity, e.g. actively holding beliefs. She argues that robust perfectionist disvalue can be satisfactorily located at neither of these two levels, and proposes that the twofold scheme should be expanded to include “outputs” of activities. It is on the level of outputs, Bradford contends, where robust perfectionist bads called “malfillment” can be found. Capacities are fulfilled whenever a right output corresponding to that capacity is reached, such as when the capacity for forming beliefs arrives at a true belief; in contrast, capacities are malfilled whenever a wrong output occurs, e.g. a false belief. This malfillment is a genuine disvalue: we are not just “faring less well”, we “fare badly”.^13^ Bradford argues that this account of perfectionist badness allows both to account for genuine perfectionist disvalue, and to explain pain as a robust bad on purely perfectionist (rather than hedonist) grounds. I won’t engage with this second part of her proposal and focus only on the general characterization of malfillment as perfectionist bad.

The malfillment view goes some way towards developing a compelling account of perfectionist badness but this proposal also has remarkable shortcomings. Given these shortcomings, perfectionism as a theory of well-being could be strengthened by an alternative account of perfectionist badness that supplements—if not supersedes—the malfillment view.

The malfillment view has two advantages. One is that it satisfies the intuition that perfectionist bads should be symmetrically opposite to perfectionist goods, just as pain is the direct opposite—and not just a lack—of pleasure in the hedonist theory. In case of malfillment, this symmetry is based on the common ground that it shares with fulfilment, namely that the capacity in both cases realizes the fitting type of output, e.g., the capacity for forming belief arrives at a belief, whether true or false. If the belief is true, then the value of realization is positive; if it is false, that the value of realization flips into the negative part of the value spectrum. The symmetry between fulfillment and malfillment thus lies in the fact that the axiological potential of arriving at the fitting type of output can be, as it were, cashed out in two different, and opposite, directions. Another advantage of this proposal is that it preserves the intuition that, insofar as we aim at a theory of well-being, any disvalue should be a disvalue for me: clearly, reaching the opposite kind of output than the one I was aiming at is a prudential disvalue.

But the malfillment view, as it stands, is vulnerable to one objection. If robust disvalue is worse than a mere privation of value, then the malfillment view would lead to the consequence that it is better not to exercise one’s capacities at all rather than having them malfilled. Consider an excellent scientist who develops an impressive and sophisticated theory, but, due to a fault of his collaborators to provide her with accurate data, this theory rests on false premises and is thus false. This scientist has malfilled her capacity for forming beliefs, and her performance has a net negative value. So, in spite of her high-level intellectual performance, the mistaken scientist fares worse than a person of mediocre intellectual capacities who holds correct but only trivial beliefs. This conclusion is quite unattractive from the perfectionist perspective, because it values incidental badness of outputs higher than inherent goodness of capacities.

To avoid this conclusion, one would have to supply one qualification for the malfillment view, namely a concession that the axiological level corresponding to activities is axiologically more potent than the axiological level corresponding to outputs, so that a mere privation on the level of exercise actually matters more for well-being than the robust badness on the level of outputs. So, malfillment is a robust bad, but it matters relatively less than whether or not one’s capacities are exercised and how good they are. This answer seems plausible: arguably, there are bads that are robust and yet trivial, as well as bads that are merely privative but severe. In some hedonist theories, an intense but short headache would arguably be a lesser evil than a life-long deprivation of pleasures of taste. With this important qualification in place, the malfillment view becomes more plausible. The activity of the mistaken scientist will contain an element of robust badness, namely the malfillment of her cognitive capacities, but her activity won't be bad all things considered, insofar as a degree of positive value that is assigned to the very exercise of these capacities is greater than the degree of negative value inherent in the bad outcome. Scientist’s activity could be bad all things considered if both the output and the exercise of her capacities is bad, e.g. when she comes to the wrong conclusion and reasons badly along the way.

One may also have misgivings about the fundamental presupposition behind the malfillment view, that is, that it is possible to separate the axiological level of outputs from the axiological level of activities. Considering the mistaken scientist case, one could point out that the badness of outputs, i.e. a mistaken scientific theory, is merely a result of the badness of scientist’s activities, insofar as the job of a scientist includes not only to develop a theory but also to double-check the correctness of her data. If the proponents of the malfillment account were to maintain that the genuine badness is to be found on the level of outputs—not activities—they would have to stipulate, in this case, that the scientific excellence in the narrow sense, i.e. the capacity for scientific reasoning on the basis of given data, can be distinguished from the scientific excellence in a broader sense, which includes also a responsible handling of the data, or even a good management of one’s research team. The badness of outputs could then be separated from the badness of activities in the former but not in the latter sense. This problem of separability is perhaps not a fatal objection against the malfillment view, but it does point in favour of an alternative account of perfectionist badness that would locate the genuine disvalue on a deeper level of the twofold scheme, i.e. on the level of activity. This is precisely what the account of badness qua vice allows us to do; in fact, this account descends on a yet more fundamental level that precedes not only outputs but also activities, namely the level of capacities. Vicious capacities are genuinely bad, and per consequence infect both activities and outputs that result from them.

My final point about Bradford’s account is that the inhibition approach to perfectionist badness is rejected too hastily. Even if we grant that an uninhibited average person fares as well (or as badly) as half-inhibited Einstein, it does not follow that Einstein’s inhibition cannot be a robust bad. For the overall amount of well-being may be identical and yet its structure may be different. Say, for instance, that the overall well-being of the uninhibited average person consists of two units of robust perfectionist goodness commensurate with the degree of perfection of their capacities. Inhibited Einsteins’s well-being consists of four positive units of goodness, given that his capacities are twice as perfect, plus two negative points of badness inflicted by his half-inhibition. The inhibition detracts two units from the four positive units conferred by the perfection of capacities, so that the overall amount of perfection will indeed end up being identical with that of the average person; but the robust badness is a genuine part of this calculation. I shall discuss in more detail in section 5 why such an inhibition could have a reasonable claim to be genuinely bad. In fact, my account of impoverishment as a perfectionist bad contains elements of the inhibition view.

## Aristotle on Things that Make Death Preferable to Life

In the first book of the *Eudemian Ethics* i.5, Aristotle discusses things “on account of which it would have been worth choosing not to be born in the first place,” if one had that choice, or even things that make “not existing at all preferable to being alive”.^14^ The latter phrase implies that these things do not merely deprive life of value, but that they make life actually worse than being dead or never having been born. Insofar as it is possible to regard Aristotle’s eudaimonistic theory as a perfectionist or proto-perfectionist theory, things under discussion are at least serious candidates for genuine perfectionist bads. The state of being dead or never having been born is the state of absolute privation; if there is something worse than this state, it must be worse, therefore, on account of a robust disvalue. In the following, I will take a closer look at the items on Aristotle’s list and discuss which of these things are promising candidates for perfectionist bads. Aristotle’s account also raises further questions; in particular, one wonders what is the relative weight of these different bads in comparison with different goods, and how much badness is needed to make a life not worth living, all things considered. Aristotle himself does not make this quite clear, and I won’t go into these difficult questions here. The bad things on Aristotle’s list can be categorized into three groups: 1) cases of extreme intellectual impoverishment, such as life reduced to pleasures of nutrition and sex or living the whole life like a child; 2) grave misfortunes, such as “disease” or “extreme of pains”; and 3) vicious character that taints life with “pleasures of a reprehensible sort”.^15^ The disvalue of grave misfortunes is typically cashed out in terms of pain; in *Nicomachean Ethics* ix.9, Aristotle lists “pain” and “vice” as two main attributes that taint the “natural goodness of being alive”. Whereas some philosophers have undertaken attempts to account for the badness of pain on perfectionist grounds let us leave pain as the eminently hedonist bad aside for now. So what remains on the list is impoverishment and vice. I submit that both these shortcomings fit the bill for robust perfectionist bads.

But what kind of bads are they, or what conception of badness do these bads entail? In the *Nicomachean Ethics*, Aristotle distinguishes between three “objects of choice”, namely fine (*kalon*), beneficial (*sumpheron*) and pleasant (*hedu*), and their contraries, i.e. shameful (*aischron*), harmful (*blaberon*) and painful (*luperon*).^16^ Among these major kinds of badness, the shameful and the harmful both come in question as possible conceptions of perfectionist badness. Indeed, we shall see that Aristotle consistently associates the state of vice with the shameful. Whereas the virtuous person aims at the “fine”, i.e. the value that defines non-instrumental goodness of a morally admirable action, such as the heroic sacrifice of one’s life on the battlefield, the vicious person, being in the shameful state of vice, is attracted to morally shameful action, such as a coward desertion. In contrast to vice, impoverishment, or special kinds of impoverishment that are particularly salient for the account of perfectionist badness, can be most plausibly understood in terms of harm. When one is deprived of certain worthwhile capacities that one had before, or when one is inhibited in their exercise, this is harmful in the sense that one is robbed of the benefit that one could have were one free of the deprivation or inhibition.

## The Badness of Vice

Insofar as perfectionism regards the development or perfection of certain human capacities as good, then virtue, understood as an excellence of these capacities, obviously counts as a perfectionist good. In contrast to the contemporary use, “virtue” or “excellence“ (*aretê*) in antiquity refers to a broad range of capacities, including purely intellectual (i.e. “theoretical” or “contemplative”) capacities and is not limited to moral goodness or excellence of character. One characteristic feature of Aristotle’s notion of vice (*kakia*) is that it is the symmetrical opposite of virtue, not a mere privation of it: it is the “opposite disposition” (*hexis enantia*), as Aristotle puts it.^17^ But why is vice the opposite of virtue, rather than a mere absence of it? Most importantly, vice presupposes the capacity of practical rationality, and for that reason neither children nor animals can be vicious. So, rather than a non-development, vice is a mis-development, where rational capacities have been developed in the wrong direction, contrary to virtue. The vocabulary of “corruption” or “perversion” (*diastrophê*) used by Aristotle captures this point well.^18^

The perversion lies in the fact that vicious humans decide rationally, by reaching a conclusion of practical syllogism, that a particular action is to be undertaken, but this decision rests on a mistaken major premise of that syllogism, or what Aristotle calls “starting points” (*archai*) of practical reasoning and action.^19^ The starting-points specify the goal or end (*telos*) of action, i.e. what is to be done or why, and as such they inform the desiderative component of “decision” (*prohairesis*). This desiderative component is distinguished from the deliberative component, whose job it is to identify the minor premises that concern how, or by what means, that goal is to be achieved.^20^ How precisely Aristotle envisages the division of labor between desiderative and deliberative component has been debated in the scholarship. For the purposes of this article, I follow the reading defended by Jessica Moss according to which Aristotle associates non-rational desires with the selection of the end and the rational deliberation with the selection of appropriate means.^21^ What is corrupted, in the state of vice, are precisely these “starting-points”. Whereas non-vicious humans, including the weak-willed persons, have healthy starting points, i.e. have genuine understanding and appreciation of the “fine” and decide to do fine actions, vicious humans are attracted to actions that are shameful (*aischron*). In contrast to weak-willed persons, who often end up acting shamefully against their decision, because they are overpowered by recalcitrant appetites, the vicious persons are not in conflict with themselves because they aim, in their very decision, at shameful actions: their desire is not divided or diminished but perverted or mis-directed.

Vice is not a mere absence of virtue: the absence of virtue is a neutral condition we are born with; vice, like virtue, is a product of our own making. This product is a result of misguided habituation, in the course of which vicious humans have learned to value bad things and disvalue good things. It is important to appreciate that vice really is a certain kind of achievement or, rather, mis-achievement: just as there is a habituation to virtue, there is a habituation to vice.^22^ You need to go through a long-term process of perverse development to become thoroughly vicious. Humans can be habituated to regarding as good either the fine or the shameful; correspondingly, their decisions are guided by desires for the fine or by desires for the shameful. The more shameful things they do, and the more gratification they find in it, the more the notion becomes ingrained in their mind that shameful things are good and thus worth pursuing. So the idea is that there is a capacity for desiring the good, or what is regarded as the good, and this capacity can be actualised in two opposite directions: either it becomes the “disposition” or “state” (*hexis*) of virtue, or the state of vice.

The state of vice also has to be distinguished from a mere error or a deficiency of reason. Vicious persons would not change their decision if someone were to explain to them that their action is shameful. For their decisions are determined by their perverted moral taste. This taste is a result of long-term mis-habituation and as such is not susceptible to rational explanation. Nor does vice entail a collapse of the rational or deliberative component of decision. In fact, there are indications that reasoning capacities are an important accomplice in this process of mis-habituation: it is due to the varieties of “rationalization, denial, confabulation, and self-deceived excuse-making” that enable vicious agents to reach the conclusions they want.^23^ So the rational capacities, too, are not diminished but rather misdirected. This is clear from his Aristotle’s distinction between practical wisdom and cleverness (*deinotês*). This is a “capacity” (*dunamis*) “which is such as to be able to do the actions that tend to promote whatever goal is assumed and to attain them”.^24^ If the goal is fine, cleverness is praiseworthy, if it is bad, cleverness amounts to unscrupulousness. Practical wisdom is not cleverness, says Aristotle, but it is not possible without this capacity (1144a30).^25^ Thus, in the state of vice both components of decision-making capacities remain fully operative. Vicious persons desire and reason as much as virtuous persons do. When it comes to the reasoning or deliberative capacities, Aristotle even thinks that their reasoning can be as well-developed, sound and logical—in the peculiar sense of purely instrumental “cleverness”—as it is in the virtuous. So, just like fulfilment and malfillment, virtue and vice have a common ground, and this is the capacity for reasoning. It is with reference to this common ground that they are symmetrically opposite. In Aristotle’s terminology, the difference between virtue and vice on the one hand, and their underlying common ground on the other hand, coincides with the terminological distinction between “capacity” (*dunamis*) and “disposition” (*hexis*). In general, it is characteristic for rational capacities that they can issue in contrary results, good or bad; “dispositions”, in contrast, are capacities that have been formed or educated so that they issue only in good or bad results: virtue as a good disposition generates only good actions, vice as a bad disposition generates only bad actions. We could say, thus, that the capacity for practical reasoning that is fully developed in “cleverness” is a kind of axiologically neutral modus operandi whose cash value depends on the kind of disposition, good or bad, that determines whether it is used well or badly.

One advantage of this conception over the idea of malfillment is that it locates the robust badness at a more fundamental level of the threefold scheme. Since badness occurs already on the level of capacity or, more precisely, state, it spoils all further levels of the threefold scheme, that is, it makes both activity and output vicious. This preempts the unpalatable eventuality that unperfected activity with incidentally right outputs could be better than perfect activity with incidentally wrong outputs. What remains untouched by vice is only the lowest level of the mere capacity of practical reasoning, regardless whether it is good or bad, i.e. a specific psychological *modus operandi*. Could it be that this *modus operandi* in and of itself, even if bad, would be more valuable than no capacity and no exercise at all? If so, the objection against the malfillment view returns again, just one level lower. But it is difficult to see why the bare fact of exercising practical rationality should have any perfectionist value if this exercise is perverted. Surely, for Aristotle the rational *modus operandi*, whether virtuous or vicious, is what enables to identify one’s activity as human, i.e. fulfilling the definition of characteristically human activities. But it is not clear in what sense can the fact that my activity can be regarded as human, rather than merely animal, be any good for me. So it is arguably the case that no activity is better than vicious activity.

So far, we have been discussing virtues that Aristotle calls virtues of character, or virtues of practical rationality. This raises the question whether the idea of vice as perversion of rationality also applies to virtues that have been called intellectual or epistemic, i.e. those that are not concerned with doing what is good but rather with knowing the truth. One may object that the peculiar feature of moral vice, namely that the perversion presupposes rather than excludes certain level of development or perfection, is unique to capacities for practical rationality, but vanishes with purely intellectual or bodily capacities. In case of bodily imperfections, at least, it is difficult to see how they could be anything else than just fallings short, deficiencies of proper functioning. But since the development of bodily capacities, in contrast to rational capacities, does not belong to universally accepted, perfectionist goods, I take it that even if it is impossible to identify their robustly bad counterparts, this does not count as a fatal consideration against perfectionist account of prudential disvalue; perhaps the fact that they cannot be bad in a robust sense could rather explain why they cannot be good in a more substantive sense that practical and theoretical capacities are.

As a matter of fact, there is little in Aristotle’s works that testifies to his concern with epistemic vices. But the rapidly emerging contemporary discourse on epistemic vice offers some useful hints towards the view that the epistemic vice has a robust disvalue that goes beyond an incapacity to discover the truth. In his introductory article into vice epistemology, Quassim Cassam argues that epistemic vices, such as closed-mindedness, rigidity or wishful thinking are character traits that “impede effective and responsible inquiry”.^26^ He gives an example of Oliver, who is deeply dedicated to a conspirational theory about 9/11. It is important to acknowledge that Oliver “is certainly an inquirer”, i.e. he invests considerable cognitive efforts to confirming this theory and vindicating its against criticism. But his inquiry is perverted in the sense that his cognitive efforts work against, rather than in favor of, the natural goal of every inquiry, i.e. to discover the truth. The more he inquires, the more convinced he becomes about his conclusions, and the further away he gets from the truth. In this respect, he is very much unlike the mistaken scientist. His epistemic vices guarantee that his capacity for inquiry is ‘malfilled’, i.e. fail to arrive at true beliefs. But, in addition to the scientist, the very activity of inquiry, in the way he pursues it, not only fails to be productive, it is counter-productive.

Likewise, in his recent contribution to the vice epistemology, Jason Baehr suggested that the possibility of mis-using or perverting the rational epistemic capacities lies in the very structure of epistemic vice. The particularly salient aspect of this structure is that all epistemic virtues have what he calls a “motivational dimension”, i.e. that the exercise of the cognitive capacities is motivated by an intrinsic concern or “love” of epistemic goods.^27^ This concern can be not fully developed, or absent, but it can also be perverted, as in the case of epistemic malevolence. Baehr considers an example of a person who has a high level of certain epistemic competences, such as open-mindedness, but he “systematically refrains from manifesting these abilities on account of his malevolence,” or perhaps even intentionally uses them to ingrain falsehoods and confusion in the minds of others. In this case, intellectual vice is a consequence of a “positively bad” epistemic motivation. Indeed, it is due to the possession—rather than absence—of some epistemic competences that this agent is vicious, and the more of them he has, the more vicious he becomes.

These remarks should suffice to give a sense of how both moral and epistemic vices contain a dimension of robust badness, namely a kind of mis-use or perversion, that cannot be reduced to a mere absence of worthwhile perfectionist capacities. In the next section, I shall turn to cases in which perfectionist bads are constituted by lacks, deprivations, inhibitions, or what I collectively call impoverishments. But even in these cases the badness is premised on the possession of certain good things, rather than on their absence. But here the underlying good is not the actual possession of some potentially good capacities but rather a potential possession of some good capacities in the future, or an actual possession of some good capacities in the past.

## The Badness of Impoverishments

Our lives may have many different kinds of lacks that pertain to the worthwhile capacities or their exercise. With regard to the best possible life, which is full of unimpeded exercise of excellent and most worthwhile capacities, these lacks make life impoverished, to a greater or lesser extent. In this section, I shall first distinguish between different kinds of impoverishments, and then, second, discuss which among these impoverishments are promising candidates for perfectionist bads.

The first distinction that comes to mind is the distinction between impoverishment on the very level of capacities and impoverishment on the level of their actualisations, i.e. what Bradford calls “inhibitions”. This distinction does not play any significant role in the following account of intrinsically bad impoverishments. Not only the inhibitions in the exercise of existing capacities but also a simple lack of capacities can be bad. Moreover, long-term inhibitions in the exercise of capacities also typically lead to the loss of these capacities.

A more salient general distinction is between privation and deprivation. When I don’t have the capacity to play chess, or when I have it but cannot exercise it, this is a real lack; other things being equal, I may be better off—depending on a specific account of perfectionist value—if I had that capacity and could exercise it. But in most cases this lack is a simple privation, rather than a deprivation. For a lack to count as a deprivation, it has to be a lack of some good which is somehow necessary for me, or which I have a reasonable claim to. We may be deprived of sleep, or food, because these count as necessities of our biologically conditioned life. But perhaps one could be deprived even of chess-playing skills in some special circumstances. Consider a talented young man who was eager to learn to play chess, but was prevented from so doing—for no good reason—by his parents. Given his talent, he had a reasonable claim to learn chess, and in that sense the fact that he ended up lacking this capacity would count as a deprivation. Another example of deprivation of capacities would be a person who was born deaf and will remain so for the rest of her life. As a consequence of this disablement, she will be deprived of some worthwhile activities, such as listening to music, that are accessible to other humans. We call this a deprivation because, being born a human, she has had a reasonable claim to having the sense of hearing just as other humans do.

There are further distinctions to be drawn within the realm of deprivation. Consider an old philosophy professor with a rapidly progressing dementia. The illness robs her of the high level intellectual capacities that she has acquired and exercised throughout her life, eventually reducing her to the mental level of an infant. We can call this kind of deprivation a loss-containing deprivation. In contrast to the person who was born deaf and thus never became deaf, the condition of the philosophy professor entails a heavy loss. She had those capacities before, but does not have them anymore.

An even more special case would be a loss-containing deprivation which, in addition, contains waste. Let us take an example of a young aspiring piano virtuoso who has dedicated all his life so far to the acquisition of his skill, sacrificing all other potentially worthwhile pursuits for its sake. Shortly before achieving his goal of becoming a virtuoso, he loses his hand. In contrast to the philosophy professor, who has been allowed to exercise her intellectual capacities throughout her life, the piano player is prevented from benefitting from what he has worked so hard towards. What is wasted are, firstly, his already very advanced piano playing skills, and, secondly, the resources that he has invested into acquiring these skills, most important of which is the time of his life.

Finally, we can consider cases of deprivation which contain waste but not necessarily loss. There are many talented children who cannot develop their talents into worthwhile perfectionist capacities. Surely, many children could easily become excellent scientists, artists or politicians if only they would be provided with supportive upbringing and appropriate education. This holds also for some adults. Many people are overwhelmed by the daily grind to develop, or even to discover, their aptitudes for worthwhile and possibly high-level preoccupations. These talents and aptitudes are not exactly lost, but they are wasted in the sense that the valuable potentialities have not been properly actualised.

Which of these states of impoverishment contain genuine bads? While it is difficult to see how the simple privation, or indeed even unqualified deprivation could be intrinsically bad, I submit that all deprivations containing loss and/or waste are bad in a way that it not merely a privation of goodness. Starting with the case of loss-containing deprivations, we can note that the badness of this condition has been discussed by Thomas Nagel and Jeff McMahan.^28^ In their view, the badness of these deprivations is indexed to one’s personal history. McMahan gives an example with two persons suffering from dementia: person A, who spent her entire life in experiencing “passive pleasures”, and person B, who devoted her life to active exercise of high-level intellectual capacities, similarly to our philosophy professor. Now, whereas an additional year of life for person A will have positive, albeit diminished value, for person B, the additional year will have “negative value”; in fact, McMahan suggests, she may well be better off dead.^29^ This comparison has a seemingly paradoxical conclusion that for person B the additional year of life is a robust bad precisely because her former life was so good. Had she not achieved such a degree of intellectual perfection before, her life would still be worth living, even in her present condition. It is consistent with the symmetrical relationship between goods and bads that it is because of the goods that we can suffer bads; the genuine badness of person’s B deprivation is derivative from the genuine goodness of the capacities she lost.

While the intuition motivated by the dementia case is quite strong, one might wish to pin down more precisely what is it about this condition that is robustly bad. I submit that this badness can be specified as a harm that the deprivation inflicts on its subject. By eating away her cognitive capacities, the dementia does not merely reduce the well-being of the professor but harms her, robbing her of the good that she would otherwise keep having. We would not say this about humans who are born deaf; of course, they are handicapped and disadvantaged by their deprivation, but not harmed. It is the possession of certain goods that makes us susceptible to harm; in fact, the greater goods we have the more susceptible to harm, or susceptible to a greater harm, we are. Still, the condition of the demented professor may not be an all-things-considered bad. She would not be harmed had she not lived an intellectually rich life; but that does not give her sufficient reasons to regret having lived this intellectually rich life. If her present unfortunate condition is the price she has to pay for her previous rich life, we may well think that that is an acceptable price to pay. Given her present condition, a continued existence may not be worthwhile, but that does not mean that she would better not have lived this life. In fact, the overall well-being of her life could still easily exceed the wellbeing of the person A.

In comparison with the demented professor, the disabled piano player is worse off. For the harm he suffers from the loss of his capacities is further aggravated by the fact that both his capacities and the time he had spent to acquire them have been wasted. He has been deprived not only of his capacities, but also of other foregone perfectionist goods that he may have likely achieved and kept were he not single-mindedly dedicated to become a piano virtuoso. Given that the piano player has not yet achieved perfection, he may in fact regret the career choice he made. Had he known that he would lose his hand, he would have chosen another kind of pursuit. The badness of his condition can be likened to the badness one suffers from losing a bet. If I bet fifty pounds on a horse that loses, the result is not only that I do not win: I lose fifty pounds. The more I bet, the more I can lose. The badness of the piano player condition is genuine because he has lost a high-risk bet. Like in his early childhood, he is now without any high-level capacities he could exercise, but his condition is different: for he is now much older and with much more limited resources to compensate for his loss.

The simple waste of capacities constitutes a harm, too. Talented children that waste their talents are harmed, and the greater their talents are the more they are harmed. What they are robbed off are not the capacities they have actually but the capacities they could easily have potentially, given their predispositions to acquire them. But the harm is comparable to the harm caused by the dementia in the sense that just as the philosophy professor has no reason to regret having had some high-level intellectual capacities in the past, the children do not have to regret the mere fact of having a reasonable prospect of acquiring some high-level capacities in the future. Rather, what is regrettable is only the loss in the former case and the waste in the latter. The case of the disabled piano-player is worse, insofar as he has reason to also regret the acquisition of his capacities, since the resources invested to acquire them could have been used to acquire and exercise other capacities.

But perhaps it is also possible to understand the badness of waste in a broader sense yet, one which would extend to the intellectually seriously impoverished lives that Aristotle deems not worth living. Insofar as you can be considered a human being, living a life reduced to passive pleasures and fully devoid of any kind of worthwhile intellectual pursuits can be regarded as a kind of waste. For, being a human, you have at least some rational capacities, but these capacities have been wasted by living such an impoverished life. However, this view is susceptible to the objection that this kind of deprivation is not necessarily bad for the person who suffers from it. If one has no aspiration to life such a life, and if one is not distressed in any way by living it, why should this way of life be harmful? This is the objection that perfectionism in general postulates a dubious gap between well-being and subjective experience, or that it is too externalist, making verdicts about well-being from a too abstract perspective that is detached from one’s individual experience: “It must be possible to specify the ultimate or fundamental conditions of my well-being without making essential reference to “other individuals, or to classes or groups of individuals”; instead, “what counts toward my well-being must depend on what I am like”.^30^

But this objection begs the question: is it at all possible to define ‘what I am like’ without making such references to other individuals or group of individuals? Arguably, my identity is partly constituted by my relationships to other human beings and by the social roles I participate in. Consider a man who is a father of three. As a father, this man is a total failure, having abused his children and wrecked their life. But in spite of this failure, he himself has managed to live a long life free from any regret or emotional distress. Would anybody say that he has fared well in his life? To do that, we would have to fully decouple his role of the father from his self. But this seems to presuppose a notion of self that is wholly disembedded from the social relationships. This is not to say that this is not possible, but only that Haybron’s objection is based on an account of the self that makes some potentially problematic assumptions. Indeed if our self turns out to be, to some extent, external to what we experience at a particular moment in time, such as involving relationships in which we participate, then vice can be obviously bad for us, insofar as it harms these relationships.

This perspective on the self can also provide some support for the possibility that some deprivations are harmful for me qua human. Being a human is not an artificial denomination that is externally imposed on me. Rather, it is a description of a peculiar set of bodily and psychological capacities, structured and arranged in a definite way, that constitute the being that I am. As such, it is an obvious referential point for my well-being. Consider the following thought-experiment. Imagine an animal in the zoo, an eagle, perhaps, that has been born and raised in a cage, never having flown or hunted in a way that free eagles do. The eagle is kept in the zoo as a representation of its natural species, which presupposes that it keeps having the attributes and capacities that define the species; and indeed it does have them, or at least most of them, so that it would still be able, theoretically at least, to acquire some of the skills that wild eagles have. The eagle is well taken care of, free from any distress and never would have thought of being able to live a different kind of life than it does. Could we say that this eagle fares well? A positive answer seems, at least, very controversial. Indeed, many would think that this is not the kind of life that eagles are supposed to have and that this condition is humiliating, despite of the fact that the bird may be quite contended with it and that it is less distressed by the hardships of wild life than it would be in the free. The reason is that it cannot live up to what it is: an eagle. If it lost the capacities that would qualify it as an eagle, i.e. cease to be a recognisable member of its species, its impoverished way of life may not be bad from the perfectionist perspective anymore. For there would not be a definite baseline for judging its well-being. But as long as it is an eagle, life unworthy of eagle constitutes a genuine disvalue. This is also the reason why many visitors feel unease in the zoo, thinking that rather than living that kind of life, it would be better for the eagle not to live at all.

Now, it is arguably the case that humans, like eagles, have some characteristic capacities that define them as members of their biological species. This is the view that Aristotle develops in the first book of the *Nicomachean Ethics.* To the extent that they lack some of these capacities, have failed to fully develop them or are prevented from their exercise, while still remaining recognisable members of the human species—they are comparable, to a greater or lesser extent, and in more and less straightforward ways, to the caged eagle. Some impoverishments, like the lack of the chess-playing skill, will be far less severe than eagle’s deprivation of flying; but others may be more serious. If some sort of intellectual pursuit or an amount of critical reflection on our life is an essential part of what makes us humans, then a life that is impoverished by lacking this dimension will be as pitiable as the life of an eagle that is deprived of flying.

This discussion about the role or function of human being as a part of our personal identity prompts a final comment about the badness of impoverishment. If a loss or waste of capacities can be robustly bad for me precisely because they are bad for the kind of subject that I am, then some of these losses will be more severe than other depending on the extent to which I identify myself with these capacities. Consider a peculiarly qualified version of the virtuoso player case, in which the piano player developed his capacity only because he was forced to do so by his family or environment, and never has embraced his career as belonging to his identity as he perceived it. When he loses this capacity, this loss does not necessarily entail a harm; in fact, he could ultimately regard this loss as a liberation that finally allows him to embark on projects that he himself is attracted to. In this case, loss is free from harm because this capacity was regarded as an external imposition that does not truly belong to the identity of this person.

Now, this case can be contrasted with a case of a deeply committed virtuoso, who regards his loss as a devastating blow that deprives his life of any direction or meaning. He has identified with his role and activity of virtuoso player to the extent that this loss results in an existential crisis in which he regards the prospect of early death as less distressing that the prospect of continued existence. In fact, his life ceases to be worth living not so much because the loss of capacities would be so overwhelmingly harmful; it might be more precise to say that the life without piano playing could not be identified as his life at all, or that, at any rate, he does not regard further existence as a continuation of his life. If this is right, then it follows that in cases such as this the loss of capacities is not necessarily worse than death but still as bad as death. For if I irreversibly lose capacities that are so centrally constitutive of my identity and life’s meaning, then I have virtually died qua the kind of person that I have been.

The acknowledgment that the degree of identification with my capacities determines the degree of harm that their loss entails raises the question whether any loss of capacities can be a genuine harm unless I perceive them as a part of my identity. And here it seems that there are two different perspectives from which a loss of a well-developed capacity can be regarded as a disvalue. From an objective perspective, one could maintain that even the uncommitted piano player has suffered a genuine harm, though she does not perceive it as such, insofar as her virtuosity counts as an objectively valuable human perfection. From a subjective perspective, one could argue that a loss of a trivial and imperfect capacity could in fact be more harmful than a loss of a respectable and perfected capacity, provided that the former plays a more significant role in how an agent construes her identity. Depending on which perspective one emphasizes, the evaluation of different losses might come out differently. But this does not worry us at this point. What we see is simply that there are two different perspectives from which the loss of capacities can constitute a genuine disvalue.

## Conclusion

I have proposed that there are some useful hints in Aristotle’s ethics that can be translated into the contemporary context and further developed so as to provide a compelling perfectionist account of robust prudential disvalue. Aristotle establishes a useful link between what is bad for us and what makes a life worse than death. If perfectionism were unable to provide a satisfactory account of robust prudential disvalue, it would be impossible to justify, on perfectionist grounds, why some kinds of lives would better not be lived. Conversely, if there are genuine perfectionist bads, they should be of such sort that it is plausible to think that they could possibly make death preferable to life. It seems that Bradford’s malfillment is just not bad enough to pass this test; it is a robust bad, perhaps, but on its own not fundamental enough to undermine the very worthwhileness of our lives. But Aristotle mentions two other shortcomings that arguably could have a more pervasively detrimental impact on the worthwhileness of our life. One is vice, or, as I have argued, perversion of our practical or theoretical rationality. Another is impoverishment, i.e. a peculiar form of deprivation that involves loss and/or waste of one’s capacities.

What vice and robustly bad kinds of impoverishment have in common is that the actual perfectionist badness they entail is, paradoxically, parasitic on an non-actual perfectionist goodness; in fact, this badness is coextensive with this goodness. I say “nonactual,” rather than “potential,” because some forms of non-actuality that are characteristic for the loss, i.e. goods had in the past, are not potential but rather not actual anymore. The badness is parasitic on goodness by inverting its value. Altogether, we have discerned three kinds of this inversion: perversion in the case of vice, where potentially good moral and cognitive capacities are misused, as well as loss and waste in case of impoverishments. What was good for us (the demented professor), what could be good for us but isn’t (the disabled piano player), or what could have been good for us but wasn't (talented children), has become the source of our ill-being.

## Footnotes

1 See Derek Parfit, *Reasons and Persons* (Oxford: Oxford University Press, 1984), p. 493.

2 See Gwen Bradford, “Perfectionism,” in Guy Fletcher ed., *The Routledge Handbook of Philosophy of Well-Being* (London: Routledge, 2016), pp. 124–134.

3 See Dale Dorsey, “Three Arguments for Perfectionism,” *Noûs* 44 (2010): 61–62 .

4 See Valerie Tiberius and Alexandra Plakias, “Well-Being,” in John Doris and The Moral Psychology Research Group eds., *Moral Psychology Handbook* (Oxford: Oxford University Press, 2010), p. 410; Daniel Haybron, *The Pursuit of Unhappiness: The Elusive Psychology of Well-Being* (Oxford: Oxford University Press, 2008).

5 See Guy Fletcher, *The Philosophy of Well-Being* (London: Routledge, 2016).

6 See Richard Kraut, *What is Good and Why* (Cambridge, MA: Harvard University Press, 2007), pp. 148–50.

7 See Shelly Kagan, “An Introduction to Ill-Being,” in M. Timmons ed., *Oxford Studies in Normative Ethics* (Oxford: Oxford University Press, 2014), p. 263.

8 See Gwen Bradford, “Perfectionist Bads,” *The Philosophical Quarterly* 71: 586–604.

9 In Bradford, op. cit. (note 2), p. 124.

10 See Daniel Russell, *Happiness for Humans* (Oxford: Oxford University Press), pp. 45–53.

11 See Anne Baril, “Virtue and Well-Being,” in Guy Fletcher ed., *The Routledge Handbook of Philosophy of Well-Being* (London: Routledge, 2016).

12 See Bradford, op. cit. (note 8), p. 591.

13 Ibid., p. 597.

14 See Aristotle, *Eudemian Ethics* 1215b. Translation according to Michael Woods, *Eudemian Ethics Books I, II and VIII* (Oxford: Clarendon Press, 1992).

15 Ibid. 1215b 22–32.

16 See *Nicomachean Ethics* 1104b 31–35. Translation according to Terence Irwin, *Aristotle: Nicomachean Ethics* (Indianapolis: Hackett, 2000).

17 See Nicomachean Ethics 1108b11–19.

18 See Rachel Barney, “Becoming Bad: Aristotle on Vice and Moral Habituation,” *Oxford Studies in Ancient Philosophy* 57 (2020): 273–308.

19 See, e.g., *Nicomachean Ethics* 1150a1–5.

20 Ibid., 1144a7–9; 1145a7.

21 See Jessica Moss, “‘Virtue Makes the Goal Right’: Virtue and Phronesis in Aristotle’s Ethics,” *Phronesis* 56 (2011): 205–261.

22 See Barney, op. cit., p. 278.

23 See Barney, op. cit., p. 300.

24 See Nicomachean Ethics, 1144a25-27

25 Ibid., 1144a30.

26 See Quassim Cassam, “Vice Epistemology,” *The Monist* 99 (2016): 164.

27 See Jason Baehr, “Epistemic Malevolence,” *Metaphilosophy* 41 (2010): 22-3.

28 See Thomas Nagel, “Death,” *Noûs* 4 (1970): 73; Jeff McMahan, *The Ethics of Killing: Problems at the Margins of Life* (New York: Oxford University Press, 2003).

29 See McMahan, op. cit., p. 175.

30 See Haybron, op. cit., p. 157.

